# Quality of websites of obstetrics and gynecology departments: a cross-sectional study

**DOI:** 10.1186/s12884-015-0537-9

**Published:** 2015-04-26

**Authors:** Günther A Rezniczek, Laura Küppers, Hubertus Heuer, Lukas A Hefler, Bernd Buerkle, Clemens B Tempfer

**Affiliations:** Department of Obstetrics and Gynecology, Ruhr-Universität Bochum, Düngelstraße 33, D-44623 Herne, Bochum Germany; Weiße Q Consulting GmbH, Dortmund, Germany; Karl Landsteiner Institute of Gynecologic Surgery and Oncology, Linz, Austria

**Keywords:** Website, Quality, World Wide Web, Obstetrics and gynecology, Score

## Abstract

**Background:**

The internet has become an easily accessible and widely used source of healthcare information. There are, however, no standardized or commonly accepted criteria for the quality of Obstetrics and Gynecology websites. In this study, we aimed to evaluate the quality of websites of Obstetrics and Gynecology departments in German-speaking countries and to compare websites nationally and internationally.

**Methods:**

We scored 672 websites from Germany (n = 566), Austria (n = 57), and Switzerland (n = 49) using the objective criteria: Google search rank (2 items), technical aspects (11 items), navigation (8 items), and content (6 items) for a 26 point score. Scores were compared nationally and internationally. Multivariable regression models assessed good quality scores (≥50% of maximum) as the dependent variables and country, academic affiliation, being member of a healthcare consortium, confessional affiliation, and content management system (CMS) use as independent variables.

**Results:**

The mean score of websites was 13.8 ± 3.3. 4.2% were rated as good (≥75% of maximum), 61.8% as fair (≥50% of maximum). German (14.0 ± 3.2) and Swiss (13.8 ± 4.0) websites scored significantly higher compared to Austrian websites (11.6 ± 2.5) (P < 0.001 and P = 0.005, respectively). Within Germany, academic had higher scores than non-academic departments (14.9 ± 3.2 vs. 13.7 ± 3.1, P < 0.001). Single institutions had higher scores compared to healthcare consortium institutions (14.1 ± 3.2 vs. 13.2 ± 2.6, P = 0.003). Departments in Northern and Southern states had higher scores compared to Eastern states (14.4 ± 3.2 and 14.2 ± 3.2 vs. 13.0 ± 3.0, P < 0.001). In multivariate regression models, all subscores (all: P < 0.001) independently predicted a website’s reaching a good quality score, with navigation subscore as strongest predictor. Affiliations were predictors for some good individual subscores, but not for others. High content subscore was associated with good Google search rank, technical aspects, and navigation subscores.

**Conclusions:**

The quality of websites of Obstetrics and Gynecology departments varies widely. We found marked differences depending on country, affiliation, and region.

**Electronic supplementary material:**

The online version of this article (doi:10.1186/s12884-015-0537-9) contains supplementary material, which is available to authorized users.

## Background

The Internet has become an easily accessible and widely used source of healthcare information for patients. Depending on the specific health problem, up to half of all affected patients seek information on the internet [1-3]. Of note, healthcare-related websites are among the most often accessed non-commercial websites [4]. Gathering information on the internet, however, can be misleading. It has been frequently found that content quality of healthcare-related websites is a problem [5-10], despite initiatives like the Health On the Net Code of Conduct (HONcode, established in 1996) [11] and the publication of the American Medical Association Internet health information guidelines in 2000 [12] and the eEurope 2002 Quality Criteria for health-related websites [13].

Looking specifically at the field of Obstetrics and Gynecology, for example, Agricola et al. demonstrated that preconception information found in a Google search is poor and inaccurate regardless whether women or health professionals performed the searches [7]. Website contents on sensitive issues such as oral contraception and abortion may be characterized by extensive misinformation, as demonstrated by recent studies [10,14]. On the other hand, other investigators have found mostly accurate web-based information on selected issues such as nausea and vomiting in pregnancy, postmenopausal osteoporosis, and female urinary incontinence [15-17]. Thus, these mixed findings highlight the need to establish quality guidelines for Obstetrics and Gynecology website contents.

Besides patients, healthcare providers also use the Internet for marketing and information purposes [18]. Today, websites are common means of marketing for all players in the healthcare market in industrialized countries, even for Departments of Obstetrics and Gynecology [19]. Obstetrics and Gynecology websites are booming, as demonstrated by a Google search (July 16, 2014) using the search terms “Obstetrics” and “Gynecology” which yielded about 10.3 M and 9.5 M hits, respectively. According to Google AdWords, 60,500 and 22,200 average monthly searches with these search terms are performed worldwide. Other keywords related to women’s health issues are searched even more often, e.g. in 2013, “pregnancy” was searched for more than 500,000 times per month in the U.S. alone [20]. In the light of this and a steadily increasing number of internet users [21], the importance of professional, well-maintained, and high quality websites is likely to increase in the future. Numerous attempts to define useful content quality indicators have been made, such as HONcode, including an associated quality label [11,22], DISCERN [23] and Brief DISCERN [24], MedCERTAIN [25], the Silberg [26] and Abbott [27] scales, and many more [28,29]. There are, however, no standardized or commonly accepted criteria for the quality of Obstetrics and Gynecology websites and to date, no studies evaluating the quality of Department of Obstetrics and Gynecology websites have been published (PUBMED search; July 16, 2014; search terms: website, internet, World Wide Web, quality, obstetrics, gynecology, score).

The PILOT Study, an online study with 1,584 participants conducted in Germany in 2011, estimated that approximately 80% of all internet users in Germany (corresponding to 40 million individuals) use the internet to obtain healthcare-related information. An additional 10 million are estimated for Austria and Switzerland [30]. Another study, based on data from approx. 20% of Germany’s population, claims that 68% of internet users are searching the Internet for health information and that women are 52% more likely to do so than men [31]. This again highlights the importance of good quality websites for women in general and those of women’s health care providers in particular. To evaluate the quality of Obstetrics and Gynecology websites we considered several indicators described in the literature. However, both DISCERN and HONcode appeared to be unsuitable for our task of evaluating websites of departments and clinics in the tightly regulated health care systems of Germany, Austria, and Switzerland [32]. A lot of HONcode principles did not apply (e.g. these sites have no ads, most items are prescribed by law), and DISCERN is considering content quality only, but not other factors such as ease of use and other factors important for website visitors seeking diverse information pertaining not primarily to treatment options but to the department or clinic itself. The Silberg score [26] was also considered, but in our opinion was too narrow for the current state of web technology and user expectations. Furthermore, department websites in Germany, Austria and Switzerland are virtually never edited by declared and/or individual authors, so a lot of the items in the previously mentioned instruments would not have been applicable. Abbott’s aesthetic criteria [33], as used in several articles on medical information website quality [34,35], was also considered and found, by itself, unsuitable.

Therefore, we designed a scoring instrument specifically for websites of Obstetrics and Gynecology departments, and used it for scoring websites in Germany, Austria, and Switzerland. Our score is based on input from the relevant target audiences and elements from other scoring systems. For example, items 3, 5, and 22 correspond to criteria of Abbott’s scale, and several other items correspond to similar items in other scales, such as interactivity/intra-site search, use of headings, presence of contact details, and dedicated sections for general and professional audiences. The aim of the study was to establish a scoring instrument for websites of Departments of Obstetrics and Gynecology, to assess the quality of such websites in three countries with a homogenous health care system, i.e. Germany, Austria, and Switzerland, and to compare the scoring results among them.

## Methods

### Study design and population

The aim of the study was to establish a scoring instrument for websites of Departments of Obstetrics and Gynecology, to assess the quality of such websites in Germany, Austria, and Switzerland, and to compare the scoring results among them. The reason for choosing these three countries was motivated by the fact that they are all German-speaking and thus websites would be more easily comparable than different language website. Furthermore, demographics and the structure of the healthcare systems in these countries are very similar [36].

The institutions’ names and addresses of the websites included in our analysis were from lists provided by the national and regional boards of Obstetricians and Gynecologists in Germany, Austria, and Switzerland. Inclusion criteria were: accessible website during the study period, clear and unambiguous attribution of the website to the corresponding institution (e.g. via imprint information).

### Design of the website score

The design of the website score followed a three-step process. In the first step, American Medical Association guidelines [12] and data in the literature [1-3,10,37] were discussed by a panel of five web professionals (with different areas of expertise and prior experience in the area of medical information-related websites) from gestaltend GmbH, Dortmund, Germany (web programmer, search engine optimization specialist, graphics designer, communication expert; this was a hired service) and Weiße Q Consulting GmbH, Dortmund, Germany (HH, business psychologist), the clinicians involved (CT, LAH, BB), and the remaining authors (GR, LK). Based on this discussion, a structured interview with 36 questions was developed, targeted at parties interested in Departments of Obstetrics and Gynecology websites (patients, especially pregnant women; patient relatives; medical students). Questions related to the probands’ internet surfing habits when seeking health care information and their preferences regarding websites of Departments of Obstetrics and Gynecology (see Additional file 1). Next, an initial design of our website score was developed, based on the results of the structured interviews with patients (N = 39), relatives of patients (N = 30), and medical students (N = 30) at the Department of Obstetrics and Gynecology, Ruhr-Universität Bochum, Germany (unpublished data). Based on the experience from evaluating a small sample of websites, the criteria of the website score were fine-tuned and finalized in a third session of the panel. The final scoring instrument for websites of Departments of Obstetrics and Gynecology consists of 27 items in four categories (Table 1). Each item/question is awarded 0 or 1 point. Note that items 8 and 9 (usage of Flash) together result in a maximum of 1 point and thus, an overall maximum score of 26 points can be reached. Furthermore, a point is always awarded for item 17 (separation of information) in case of purely gynecologic or purely obstetric clinics, and for item 27 (birth numbers) in case of purely gynecologic clinics. The four categories are: (a) Google search rank (2 items). This evaluates the results of a Google search for “Frauenklinik city”, where “city” is replaced with the actual city of the respective department; “Frauenklinik” is the most common German term for “obstetrics/gynecology clinic”. We only considered Google as search engine, because of its dominance in the search engine market, especially in Germany (94% market penetrance in Dec 2014) [38]. (b) Technical aspects (11 items). The focus here lies within technical implementation details of the website with focus on compatibility with different viewing devices, including screen readers for viewers with visual impairments. (c) Navigation (8 items). Here, usability aspects such as easy navigation, accessibility of relevant information (based on results of the structured interviews) and intra-site search functionality are considered. (d) Content (6 items). Here, the availability of selected additional information of high importance for the different target audiences of the website, such as emergency numbers, (in depth) medical information, number of births per year, or images of team members are assessed.

**Table 1:** **Website-score using 27 test items in four categories for a maximal score of 26 points**

		**Overall N = 672**	**German N = 566**	**Austrian N = 57**	**Swiss N = 49**
	**Total website-score (26)**	13.8 ± 3.3	14.0 ± 3.2 ***	11.6 ± 2.6	13.8 ± 4.0 ^^
	**Google search rank (2)**	1.3 ± 0.8	1.4 ± 0.8 ***	0.8 ± 0.9	1.3 ± 0.8 ^^
1	When performing a Google search, is a link to the department (or the hosting institution) listed on the first page of results? (Y = 1, N = 0)	528 (78.6%)	467 (82.5%) ***	23 (40.4%)	38 (77.6%) ^^^
2	Is there a direct link to the departments starting page within the first page of Google results? (Y = 1, N = 0)	362 (53.9%)	317 (56.0%) **	19 (33.3%)	26 (53.1%)
	**Technical aspects (10)**	4.6 ± 1.4	4.6 ± 1.4	4.7 ± 1.4	4.4 ± 1.4
3	Do individual sub-pages have specific and meaningful titles? (Y = 1, N = 0)	607 (90.3%)	510 (90.1%)	53 (93.0%)	44 (89.8%)
4	For individual sub-pages, is a specific and meaningful description provided via the META/description tag? (Y = 1, N = 0)	212 (31.5%)	183 (32.3%)	14 (24.6%)	15 (30.6%)
5	Are the semantic HTML tags for headings (<H1>, <H2>, …), paragraphs (<P>), and tables (<TABLE>) used appropriately? (Y = 1, N = 0)	500 (74.4%)	420 (74.2%)	46 (80.7%)	34 (69.4%)
6	For all content-related images, are meaningful descriptions provided in the ALT-attribute of the < IMG > tags? (Y = 1, N = 0)	203 (30.2%)	178 (31.4%)	14 (24.6%)	11 (22.4%)
7	Does the website provide useful/useable information even when CSS, JavaScript, and images are disabled or missing? (Y = 1, N = 0)	546 (81.3%)	458 (80.9%)	48 (84.2%)	40 (81.6%)
8	Does the website make use of Flash plug-in? (Y = 0, N = 1)	642 (95.5%)	540 (95.4%)	55 (96.5%)	47 (95.9%)
9	In case the website uses Flash, are alternatives offered for website visitors using Flash-incapable devices, or does the website at least make it obvious to visitors that they miss out on content? (Y = 1, N = 0)	20 (3.0%)	19 (3.4%)	0 (0.0%)	1 (2.0%)
10	Is the layout of the website responsive (i.e. does it adapt do varying screen sizes), or is a separate version for mobile devices available? (Y = 1, N = 0)	59 (8.8%)	45 (8.0%) °°	3 (5.3%)	11 (22.4%) ^
11	Does the website offer means to adjust (increase) the text size without compromising the functionality of the website? (Y = 1, N = 0)	245 (36.4%)	209 (36.9%)	25 (43.9%)	11 (22.4%) ^
12	Does the website offer means to adjust (increase) the contrast of textual information for visitors with visual impairments (Y = 1, N = 0)	13 (1.9%)	11 (1.9%)	2 (3.5%)	0 (0.0%)
13	Does the website offer information in one or more additional languages? (Y = 1, N = 0)	54 (8.0%)	45 (8.0%)	7 (12.3%)	2 (4.1%)
	**Navigation (8)**	5.2 ± 1.4	5.3 ± 1.4 ***	4.5 ± 1.3	5.3 ± 1.4 ^^
14	Does the department’s website have a dedicated starting (welcome) page? (Y = 1, N = 0)	643 (95.7%)	541 (95.6%)	54 (94.7%)	48 (98.0%)
15	Does the website provide a (consistently accessible) menu structure for navigating the department’s sub-pages? (Y = 1, N = 0)	545 (81.1%)	470 (83.0%) ***	33 (57.9%)	42 (85.7%) ^^
16	Does the website provide a (working) search facility? (Y = 1, N = 0)	554 (82.4%)	457 (80.7%) °	50 (87.7%)	47 (95.9%)
17	Are there dedicated subsections of the website for obstetric and general gynecologic patients? (Y = 1, N = 0)	384 (57.1%)	338 (59.7%) ***	14 (24.6%)	32 (65.3%) ^^^
18	Does the website contain a subsection directed specifically at referring physicians? (Y = 1, N = 0)	69 (10.3%)	54 (9.5%) °	5 (8.8%)	10 (20.4%)
19	Is there a (direct navigational) link to information about the department’s staff? (Y = 1, N = 0)	519 (77.2%)	444 (78.4%)	41 (71.9%)	34 (69.4%)
20	Is (department-specific) contact information easily accessible? (Y = 1, N = 0)	616 (91.7%)	528 (93.3%) °	49 (86.0%)	39 (79.6%)
21	Does the website provide access to information on how to reach the clinic (by car or public transportation)? (Y = 1, N = 0)	175 (26.0%)	158 (27.9%)	10 (17.5%)	7 (14.3%)
	**Content (6)**	2.6 ± 1.3	2.7 ± 1.3 ***	1.7 ± 1.1	2.8 ± 1.8 ^^
22	Does the website feature content-related visual impressions? (Y = 1, N = 0)	532 (79.2%)	461 (81.4%) * °	38 (66.7%)	33
23	Does the website contain (prominently displayed) emergency information? (Y = 1, N = 0)	167 (24.9%)	136 (24.0%) *** °°°	1 (1.8%)	30 ^^^
24	Are photos of the medical team (physicians, nurses) available? (Y = 1, N = 0)	569 (84.7%)	500 (88.3%) *** °°°	40 (70.2%)	29
25	Does the website provide detailed information about the offered medical service spectrum? (Y = 1, N = 0)	200 (29.8%)	167 (29.5%) °	10 (17.5%)	23 ^^
26	Does the website provide an up-to-date news/events schedule? (Y = 1, N = 0)	90 (13.4%)	75 (13.3%) * °°	1 (1.8%)	14 ^^^
27	Does the website provide information on births per year? (Y = 1, N = 0)	207 (30.8%)	193 (34.1%) ** °°	7 (12.3%)	7

The points awarded for each item/question are indicated (Y = Yes, N = No). Subcategory cumulative results and Website-Score are given as mean ± standard deviation; for individual score items the number of sites awarded with a point is shown (percentages in parentheses). Levels of statistical significance for differences are marked with 3 (P < 0.001), 2 (P < 0.01), or 1 (P < 0.05) symbol(s): * = German vs. Austrian, ^ = Swiss vs. Austrian, ° = German vs. Swiss websites.

### Website evaluation

Two authors (LK, GR) evaluated the websites with the help of a custom-programmed (GR) website analysis tool (a Microsoft . NET/WinForms application that takes a list of web addresses as input and then displays the individual websites inside the Firefox web browser via Selenium WebDriver (http://www.seleniumhq.org). For each website, the evaluating person was presented with a series of specific questions and a corresponding choice of answer buttons and/or input fields. The software documented each step of the process, made screen shots of the websites, and, where possible, facilitated the decision making by automatically extracting information from the HTML source and presenting it to the evaluating person (mostly for items under “technical aspects”). Finally, the results were exported to an Excel spreadsheet. Websites were tagged as academic and/or denominational and/or belonging to a healthcare consortium based on information from the websites (e.g. parent corporate websites, statements, imprints).

### Statistical analysis

Scores were compared nationally for Germany (academic vs. non-academic departments, healthcare consortiums vs. single institutions, whether the department operated in a denominational setting vs. not, and by region) and internationally (Germany vs. Austria vs. Switzerland). Within Germany, comparisons were made between Eastern and Northern + Southern states, based on the historical division of Germany into the Federal Republic of Germany (FRG; Northern and Southern states) and the German Democratic Republic (GDR; Eastern states) until 1989. Within the former FRG we compared Northern and Southern states based on well-known differences in economic productivity, income, and education in favor of Southern states [39]. Northern states are North Rhine-Westphalia, Saarland, Rhineland Palatinate, Hessen, Lower Saxony, Schleswig-Holstein, Bremen, and Hamburg; Southern states are Baden-Wurttemberg and Bavaria; Eastern states are: Mecklenburg-West Pomerania, Brandenburg, Saxony, Saxony-Anhalt, Thuringia, and Berlin. Categorical variables were analyzed by χ^2^-test and continuous variables were compared using the Mann–Whitney rank sum test with a significance level of 0.05. We performed a multivariate regression analysis with good quality overall and sub-scores (>50% of the maximum score) as the dependent variables and country, Google search rank, technical aspects, navigation, and content scores as well as various affiliations and content management system (CMS) usage as independent variables. Excel 2003 (Microsoft) was used to prepare the raw data, SigmaPlot 12.5 (Systat Software) was used for statistical evaluation and data visualization. Adobe Illustrator was used for final figure assembly.

## Results

### Website quality differs between Germany/Switzerland and Austria

**Figure 1:**
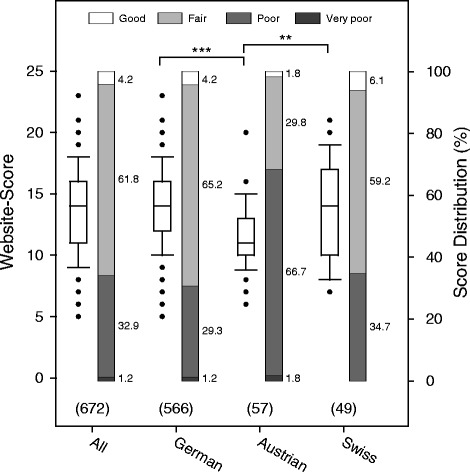
Website-scores by country, shown as box plots where the boundary of the box closest to zero indicates the 25th percentile, the line within the box marks the median, and the boundary of the box farthest from zero indicates the 75th percentile. Whiskers above and below the box indicate the 90th and 10th percentiles; points represent outliers. Stacked bars show the score distribution when categorized as good, fair, poor, or very poor (corresponding to scores ≥75%, ≥50%, ≥25%, and <25% of the maximum score, respectively). Numbers to the right of the stacked bars give the corresponding percentages. Numbers in parenthesis denote the number of websites represented by the graphs. Statistically significant differences between groups are indicated: ***P < 0.001, **P < 0.01.

In a cross-sectional evaluation, we scored 672 websites from Germany (n = 566), Austria (n = 57), and Switzerland (n = 49) drawn from the World Wide Web (WWW) between May 27 and July 11, 2014. The mean score of all 672 websites was 13.8 ± 3.3 (SD). Table 1 shows the mean overall scores and the mean subscores for Google search rank, technical aspects, navigation, and content for German, Austrian, and Swiss websites. 28/672 (4.2%) websites were rated as good, i.e. reaching at least 75% of the maximal achievable score. 415/672 (61.8%) websites reached at least 50% of the score and were rated as being of fair quality. Of the remaining websites, 221/672 (32.9%) were rated as poor (at least 25% of the maximum score) and 8/678 (1.2%) as very poor (less than 25% of the maximum score). Figure 1 shows a box plot of all scored websites broken down by country of origin, demonstrating a wide range of scores within all three investigated countries. Significant differences of mean scores were found comparing German, Austrian, and Swiss websites. German websites (14.0 ± 3.2) and Swiss websites (13.8 ± 4.0) scored significantly higher compared to Austrian websites (11.6 ± 2.5) (P < 0.001 and P = 0.005, respectively), whereas there was no significant difference between German and Swiss websites (P = 0.84).

The proportions of good quality and fair quality German, Austrian, and Swiss websites were 24/566 (4.2%) and 369/566 (65.2%), 1/57 (1.8%) and 17/57 (29.8%), and 3/49 (6.1%) and 29/49 (59.2%), respectively (see stacked bars in Figure 1).

### Affiliation is a predictor for website quality in Germany

**Figure 2:**
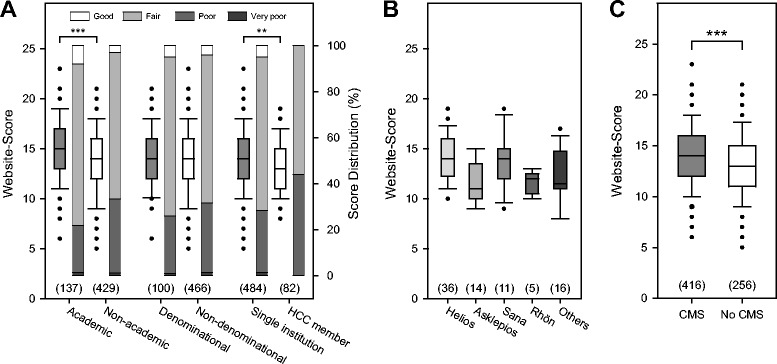
Website-scores by affiliation **(A)**, different healthcare consortia **(B)**, and CMS usage **(C)**. Only data from Germany is considered. See legend to Figure 1 for an explanation of graph elements. Statistically significant differences between groups are indicated: ***P < 0.001, **P < 0.01.

Table 2 shows the mean overall scores and the mean subscores for German websites broken down by affiliation and region, and Figure 2A shows scores by affiliation (box plots) and quality proportions (stacked bars). Within Germany, academic departments put more emphasis on website quality compared to non-academic departments (14.9 ± 3.2 vs. 13.7 ± 3.1, P < 0.001). This was also true for single institutions as opposed to those integrated in a healthcare consortium (HCC) (14.1 ± 3.2 vs. 13.2 ± 2.6, P = 0.003). There was, however, no statistically significant difference in scores between denominational vs. non-denominational institutions (14.3 ± 2.9 vs. 13.9 ± 3.2, P = 0.31). Institutions from Northern and Southern states had higher mean website scores compared to institutions from Eastern states (14.4 ± 3.2 and 14.2 ± 3.2 vs. 13.0 ± 3.0, P < 0.001). Figure 2B shows scores of HCC where at least the websites of 5 member departments have been evaluated. While the intra-HCC score variation was smaller (mean SD 2.2 vs. 3.3, P = 0.03), the range of website quality was still considerable within members of the same HCC.

**Table 2:** **Mean overall website scores and mean sub-scores of websites broken down by affiliation, region, and usage of an established content management system (CMS)**

**Score**						
	**N**	**Overall**	**Google search rank**	**Technical aspects**	**Navigation**	**Content**
**Affiliation**						
Academic vs.	137	14.9±3.2	1.6±0.7	4.7±1.5	5.7±1.3	2.9±1.3
Non-Academic	429	13.7±3.1***	1.3±0.8***	4.6±1.4	5.2±1.4**	2.6±1.2**
Denominational vs.	100	14.3±2.9	1.2±0.9	4.7±1.3	5.6±1.2	2.8±1.2
Non-denominational	466	13.9±3.2	1.4±0.7*	4.6±1.5	5.2±1.5*	2.7±1.3
Healthcare Consortium vs.	82	13.2±2.6	1.3±0.7	4.7±1.1	5.0±1.3	2.1±1.1
Single Institution	484	14.1±3.2**	1.4±0.8	4.6±1.5	5.3±1.4	2.8±1.2***
**Region**						
North	293	14.4±3.2***	1.4±0.8	4.6±1.4	5.5±1.4***	2.9±1.2***
South	138	14.2±3.2***	1.3±0.8	4.8±1.4	5.3±1.4**	2.8±1.3**
East	135	13.0±3.0	1.4±0.7	4.6±1.5	4.7±1.4	2.3±1.2
**CMS**						
CMS Usage vs.	416	14.2±3.1	1.3±0.8	4.9±1.3	5.3±1.3	2.7±1.3
No CMS Usage	256	13.1±3.4***	1.3±0.8	4.2±1.6***	5.0±1.6*	2.5±1.3

Values are given as mean ± standard deviation. Significance levels are indicated: ***P < 0.001, **P < 0.01, *P < 0.05 (Regions: North vs. East and South vs. East; no statistically significant difference in any category for North vs. South). For affiliations and regions only websites from Germany were considered.

### Content management system usage correlates with higher quality scores

416/672 (61.9%) websites in Germany, Austria, and Switzerland were identified to be built upon established content management systems (CMS; Table 2 and Figure 2C). These websites scored higher than websites without underlying CMS (14.2 ± 3.1 vs. 13.1 ± 3.4, P *<* 0.001). TYPO3 was by far (76.4%) the most commonly used CMS, followed by Joomla! (6.7%). All others represented less than 2%. ANOVA on ranks of the overall scores of websites using the top 5 CMS (at least used in ≥ 7 websites) showed no differences in the median scores among them (P = 0.240).

### Multiple regression analysis

In a multivariable logistic regression model, Google score (odds ratio [OR] 7.5, 95% confidence interval [CI] 4.1 to 13.6, P < 0.001), technical score (OR 14.1, 95% CI 8.1 to 24.7, P < 0.001), navigation score (OR 106.9, 95% CI 33.5 to 341.0, P < 0.001), and content score (OR 13.7, 95% CI 8.0 to 23.5, P < 0.001), but not country (OR 0.7, 95% CI 0.5 to 1.1, P = 0.14) were independent predictors of a website’s reaching a good website score, i.e. at least 50% of the maximum score. In addition, we calculated which items predicted a good overall score, a good Google search rank score, a good technical score, a good navigation score, and a good content score, with good defined as ≥50% of the maximum score (Table 3).

**Table 3:** **Multiple regression analysis (overall, 672 websites)**

**Variables:** Dependent > ∨ Independent	Good Overall Score	Good Google Search Rank Score	Good Technical Aspects Score	Good Navigation Score	Good Content Score
Google search rank score	-	-	n.s.	n.s.	P = 0.025
					OR 1.28 (1.03-1.59)
Technical aspects score	-	n.s.	-	n.s.	P = 0.005
					OR 1.19 (CI 1.05-1.35)
Navigation score	-	n.s.	n.s.	-	P < 0.001
					OR 1.91 (CI 1.65-2.21)
Content score	-	P = 0.006	P = 0.036	P < 0.001	-
		OR 1.28 (CI 1.07-1.53)	OR 1.16 (CI 1.01-1.21)	OR 2.32 (CI 1.81-2.97)	
Academic affiliation	P = 0.002	P = 0.012	n.s.	n.s.	n.s.
	OR 2.00 (CI 1.03-3.08)	OR 2.05 (CI 1.17-3.57)			
Single institution	P = 0.043	P = 0.033	n.s.	n.s.	P < 0.001
	OR 1.61 (CI 1.02-2.56)	OR 0.49 (CI 0.25-0.94)			OR 2.87 (CI 1.71-4.81)
Confessional affiliation	n.s.	P = 0.001	n.s.	n.s.	n.s.
		OR 0.45 (CI 0.28-0.73)			
CMS usage	P < 0.001	n.s.	P = 0.001	P = 0.009	n.s.
	OR 1.82 (CI 1.30-2.54)		OR 1.76 (CI 1.28-2.44)	OR 1.99 (CI 1.19-3.32)	

“Good” means at least 50% of the total possible points in the respective category. OR = Odds ratio; CI = 95% confidence interval; n.s. = not significant.

## Discussion

In this cross-sectional study, we found that the quality of websites of Obstetrics and Gynecology departments varies widely depending on country, affiliation, and region. To our knowledge, this is the first study to evaluate the quality of department websites in the field of Obstetrics and Gynecology according to objective criteria. While 62% of the investigated websites were found to be of good quality, with good quality defined as ≥50% of the maximum score, only few (4.2%) websites reached a good score result (defined as ≥75% of the maximum score), and none were rates as excellent (≥90%). This suggests that the overall quality of current websites of Departments of Obstetrics and Gynecology in the evaluated countries is low and that in fact all departments have an opportunity to improve their internet-based communication with current and potential patients.

Within Germany, we found marked differences between Eastern states (representing the former GDR) and Northern and Southern states (representing the former FRG) regarding all major items, i.e. Google search rank, technical aspects, navigation and content. This is interesting, because it demonstrates that historical divisions are still present in at least some aspects of the healthcare system despite >25 years of German unification [40]. However, within all German states, other major influential factors were identified. Specifically, we found that academic vs. non-academic institutions and single institutions vs. members of HCC consistently scored higher on the website score. This shows that both intellectual/academic as well as economic factors have a direct impact on the quality of Obstetrics and Gynecology department websites. This is consistent with the results of Huang et al. who assessed U.S. fertility clinic websites and found that university-affiliated sites performed significantly better. For example, university-affiliated centers were more likely to include ownership and affiliation information, web contents were significantly easier to distinguish from advertisements, reference sources were significantly easier to identify and more likely to indicate relevant financial disclosures and to include a search function [37]. Several other authors, however, did not find marked differences when comparing Academic and Non-Academic websites in areas such as bipolar disorder, social phobia, pathological gambling, and substance abuse. In these studies, however, any form of web-based health information was investigated and not only hospital departments as in our case [34,35].

In a multivariate regression analysis, several other predictors of a good overall score as well as good subscores were identified. For example, we found that being an academic site conferred the highest likelihood for reaching a good overall score and a good Google score. Being a singular institution as opposed to being a member of a HCC best predicted a good content score. This shows that there is no single item universally predicting a good website. Some characteristics predict some quality aspects, but not others.

CMS use was a reliable predictor for reaching good technical and good navigation scores. This is not surprising as items in these subscores correspond to essential features and benefits usually provided by a CMS: separation of a website’s content, its structure and its visual design; provision of a navigation system to access content; solid and standards-conforming underlying web framework. Only 8.8% of websites supported mobile browsers, indicating that most websites were designed without recognition of the strongly increasing use of mobile devices to access the Internet [41]. As our data show, CMS use, however, does not automatically mean good content or good Google search rank. There is clearly an opportunity to improve search ranks, especially for Austrian websites, and the need for medical information websites in general to carefully invest in search engine optimization is underscored by our data and has also been recognized by others [42].

Websites are an important means of information [31] and thus a direct-to-patient marketing opportunity for Departments of Obstetrics and Gynecology. The results of our study suggest that the potential to improve Obstetrics and Gynecology websites is considerable, especially in selected countries, regions, health consortium clinics, and non-academic departments, which tended to reach lower quality scores in our study. Although there is general agreement on what makes a good website, regarding both technical and content-related aspects, the level of practical implementation is obviously heterogeneous in Obstetrics and Gynecology websites. The website score presented in our study may be a helpful tool for some Departments of Obstetrics and Gynecology (clinic directors and other healthcare professionals, as well as for the respective economic and marketing units) to evaluate the quality of their own website, to benchmark their website against those of local and regional competitors, and to identify areas of possible improvement, especially because our score was designed by taking the website users’ perspectives into account. The strengths and weaknesses of a given website can be easily identified using the subcategories Google search rank, technical aspects, navigation, and content.

The strength of our study lies in the large sample of websites we have included in our analysis. However, our study has limitations. First, we only assessed websites from Obstetrics and Gynecology departments in German-speaking countries, i.e. Germany, Austria, and Switzerland. The website quality in these countries may not be representative for other industrialized countries. Therefore, our data may over- or underestimate the general website quality of Obstetrics and Gynecology departments in Western industrialized countries. Regarding the external validity and clinical implications of our study, the data therefore have to be interpreted with caution. Second, other developed countries and regions such as Japan or South-east Asia may put more emphasis on items other than those typical for Western nations due to cultural differences. This may result in different scores. A culturally-sensitive approach is needed when applying the website score published in this study to institutions in non-Western countries.

## Conclusion

In summary, the data presented in this study provide evidence that the quality of websites of Departments of Obstetrics and Gynecology varies widely both within countries and internationally. Also, selected affiliation characteristics such as non-academic institution and being part of a healthcare consortium were associated with lower scores. The mean overall score of all websites was low indicating a significant potential for improvement of Obstetrics and Gynecology websites in the majority of cases. Our data may be used by healthcare providers to evaluate their own website, identify areas of improvement, and to objectively compare their own website with local and national competitors. We hope that these data will contribute to the improvement of the quality of Obstetrics and Gynecology websites, since websites have become an important source of information for women in need of specialty care.

## Additional file

Additional file 1:
**Questionnaire.**
