# Supplementary material for: Quality of websites of obstetrics and gynecology departments: a cross-sectional study
Source: BMC Pregnancy Childbirth. 2015 Apr 26;15:103. doi: 10.1186/s12884-015-0537-9 (PMC4527247; doi:10.1186/s12884-015-0537-9)
Supplement: Additional file 1: — Questionnaire. [file 12884_2015_537_MOESM1_ESM.pdf]

Rezniczek et al., Additional file 1: Questionnaire

Inclusion criteria: patients, patient relatives, or medical students who have regular internet access; proband type (patient/pregnant patient/relative/student), sex, and age was recorded.

1. Have you previously used the Internet to obtain information about medical topics? [Yes/No]
2. Have you ever obtained/looked for pregnancy-related information on the Internet? [Yes/No]
3. What information would you expect to find on the website of the Department of Obstetrics and Gynecology of <Hospital Name>? [Box for free answer]

Questions 4-32: **How important for you is/are ….** [1 (not important at all) – 7 (very important)]

4. Contact details?
5. Information on how to get to the clinic (individual / public transportation, maps, etc.)?
6. Parking space?
7. Presentation of the team members?
8. Visual impressions in general?
9. Photos of the physicians?
10. Photos of the clinic hospital?
11. Videos (virtual tour)?
12. Information regarding patient registration procedures?
13. Information on consultation hours?
14. Floor maps of the hospital / clinic?
15. Clear separation of information for patients and professionals (info domains)?
16. Access to as many topics as possible from a central point (start page, menu)?
17. Site-specific search functionality?
18. Information on optional psychological counseling?
19. Information about research and education?
20. Information of certificates?
21. Access to press releases?
22. Information on ongoing medial studies?
23. Images/visual impressions of the delivery ward?
24. Availability of a baby gallery?
25. Clear separation of gynecologic from obstetrics concerns?
26. Specific info for expectant mothers?
27. Information on breast feeding?
28. Contact details of midwives for home care?
29. Information regarding birth?
30. Information about various gynecologic procedures?

31. Information about chemotherapy?
32. Information about radio-therapy?
33. What are the topics/items you would consider essential for any clinic website? [Box for free answer]
34. What information would you specifically expect to be present on the website of an Obstetrics and Gynecology clinic but not on that of any other type of clinic? [Box for free answer]
35. Have you visited the website of <Ob/Gyn Clinic>? [Yes/No]
36. [If 35 was Yes] How did you arrive at the website of the Department of Obstetrics and Gynecology [via hospital site/used a search engine/direct entry of website address/don’t know/other]
